# Silicon Microthermocycler for Point-of-Care Analytical Systems: Modeling, Design, and Fabrication

**DOI:** 10.3390/mi15111325

**Published:** 2024-10-30

**Authors:** Borut Pečar, Aljaž Zadravec, Danilo Vrtačnik, Matej Možek

**Affiliations:** Laboratory of Microsensor Structures and Electronics, Faculty of Electrical Engineering, University of Ljubljana, Tržaška 25, SI-1000 Ljubljana, Slovenia; aljaz.zadravec@fe.uni-lj.si (A.Z.); danilo.vrtacnik@fe.uni-lj.si (D.V.); matej.mozek@fe.uni-lj.si (M.M.)

**Keywords:** microthermocycler, thermal capacitance, thermal conductance, thermal time constant, numerical simulation, analytical model, POC analytical systems, PCR, reaction chamber

## Abstract

A four-tether silicon microthermocycler for point-of-care PCR analytical systems is proposed. Substituting the commonly employed platinum with titanium in the fabrication of thin film resistance temperature detectors and heaters enabled the realization of a smaller device without compromising temperature accuracy or increasing heater lead power losses. The device was extensively analyzed through analytical modeling and FEM numerical simulations using a 3-D thermo-mechanical simulation model in COMSOL. Numerical simulations revealed that the four-tether design provides a 460% improvement in mechanical strength and a 57% reduction in the thermal time constant compared with a similar three-tether design, with a trade-off of a 22% increase in heat losses. Detailed structural and thermal analyses of crucial design parameters guided the optimization of the final geometry, leading to the successful fabrication of prototypes. It was shown that the current of 60 mA was sufficient to heat the fabricated solid and hollow silicon structure to 132 °C and 134 °C in 10 s for an applied heater power of 510 mW and 525 mW, respectively.

## 1. Introduction

In recent years, there has been a growing demand for rapid and reliable diagnostic tools in various fields, ranging from healthcare to environmental monitoring [1]. Among these tools, ThermoCyclers (TCs) play a crucial role in performing Polymerase Chain Reactions (PCRs), enabling the amplification of DeoxyriboNucleic Acid (DNA) sequences for various applications, including disease diagnosis, genetic testing, and forensic analysis [2].

During every amplification cycle, the DNA sample doubles the previous amount. The process involves three major steps. The first is denaturation, which occurs at around 95 °C of temperature. Then comes the process of annealing, which occurs at around 55 °C of temperature, and finally, extension occurs at a medium temperature of around 72 °C. These repeating processes result in a few billion copies of the sample DNA in around 30–40 complete cycles [3].

Traditional TCs have limitations in terms of size, cost, and portability, hindering their integration into Point-Of-Care (POC) systems. The large thermal mass of the system results in high power requirements and slow heating and cooling rates [4].

To overcome these limitations, researchers have turned their attention to developing miniaturized TCs using Micro-Electro-Mechanical System (MEMS) technologies. MEMS technology offers the potential to create compact, cost-effective, and highly efficient TCs, revolutionizing POC diagnostics and bringing PCR-based tests closer to the patient’s bedside [5]. With continued advancements in MEMS fabrication technologies, coupled with innovative designs and integration with complementary technologies, the future of diagnostic testing holds great promise in terms of improved accessibility, accuracy, and efficiency, ultimately leading to better healthcare outcomes.

One of the key advantages of MEMS-based TCs lies in their ability to reduce the required sample and reagent volumes, resulting in decreased costs and improved sensitivity. By leveraging microfluidic channels and integrated heaters, these devices can accurately control temperature profiles and rapidly alternate between heating and cooling cycles (typically 15–80 °C s^−1^), enabling faster PCR reactions compared with conventional benchtop TCs [6]. The typical power consumption of these devices at 94 °C (denaturation temperature) is well below 2 W [6]. Moreover, the miniaturized nature of MEMS-based TCs allows for multiplexing, enabling parallel amplification of multiple DNA targets within a single device [7].

To ensure the rapid heating of the TC reaction chamber, numerous approaches have been reported, including induction heaters [8], laser-induced heaters [9], Peltier elements [10], IR heaters [11], and thin film heaters. A number of studies have demonstrated that thin film heaters provide excellent heating/cooling rates, leading to a rapid PCR amplification of a gene fragment [12]. To analyze the thermal response of thin film heaters, researchers applied various simulation models (in Coventor Ware™, ANSYS, FLUENT, etc.) [13,14]. Such studies were helpful in optimizing the size, shape, and position of thin film heaters, resulting in uniform temperature distribution on the microchip.

An SU-8 based TC chip was presented by J. El-Ali et al. [6]. The chamber was manufactured on a 1 mm thick glass substrate with an integrated thin film platinum heater and sensors elements. With heating and cooling rates of up to 50 and 30 °C s^−1^, respectively, the performance of the chip was comparable to some silicon micromachined PCR chips presented in the literature.

A polycarbonate TC chip was proposed by [15]. The PCR amplification chamber could hold 25 μL and comprised two 0.05 mm thick polycarbonate films and two rubber plugs. The chip body was machined on a CNC, and the films were cut on a laser cutter. The maximum heating and cooling rates for the 25 μL solution reached 24.12 °C s^−1^ and 25.28 °C s^−1^, respectively.

A glass TC chip was presented by [16]. The microheater was fabricated by patterning gold electrodes on a glass substrate, which carried out the thermocycling process. The proposed device demonstrated high-quality amplification comparable to conventional PCR devices. Another glass TC chip was proposed by [2]. Their thermocycler consisted of a compact glass microchip that could hold a 2 μL PCR sample and a surface-mounted chip resistor. The assembled thermocycler heated the sample at a maximum rate of 28.8 °C s^−1^.

In the field of POC systems, silicon-based TCs, in particular, have gained significant attention [17,18,19]. Silicon, with its excellent thermal conductivity, mechanical stability, and compatibility with microfabrication processes, provides an ideal platform for developing highly integrated and efficient TCs. By employing MEMS fabrication techniques, such as deep reactive ion etching (DRIE) and thin film deposition, silicon TCs can be designed with precise control over temperature, uniform heat transfer, and rapid thermal cycling capabilities [20,21].

Several TCs comprising thin film heaters were reported in the literature. A glass channel with a meandering design that guides the reaction mixture through the three temperature zones was proposed by Ivonne Schneegaß et al. [22]. The TC chip comprised a reaction channel etched into a glass substrate and a cover substrate made of silicon and equipped with thin film platinum heaters and sensors for temperature control. The energy consumption of the chip for a 35 min PCR process amounted to less than 0.012 kWh. Unfortunately, the authors did not measure the average heating and cooling rates.

An interesting approach to implementing a battery-powered portable silicon TC was reported by Junyao Jie et al. [23]. The chip contained four thin film aluminum heaters and four RTDs to provide thermal uniformity and accurate monitoring. The disposable reaction chips with two chambers were fabricated from silicon, silicone rubber, and a quartz plate. The average heating rate was 32 °C s^−1^, and the average cooling rate was −7.5 °C s^−1^.

To minimize thermal losses in silicon TCs, a reaction chamber should be thermally isolated from the silicon substrate. Two different approaches were reported in the literature, both employing separating tethers that thermally separate the central part of the TC from the substrate. The first approach, which was reported by Daniel, J. H. et al. [24], involves separating tethers fabricated from silicon nitride. In this case, the starting material was a 400 µm thick silicon wafer coated on both sides with a low-stress silicon nitride layer. Afterward, an aqueous KOH anisotropic wet etching of the silicon through the wafer was employed. The reported thermal time constant for the chamber filled with 1.5 µL of water and 1 µL of silicone oil was below 0.4 s. The second approach to reduce thermal losses includes silicon-separating tethers. For this approach, a thorough wafer etching of uncoated silicon is required, which poses a significant challenge in terms of establishing an appropriate fabrication process. One of the best examples of using this approach is by Neuzil et al. [25]. Their three-tether micro TC structures yielded a heating rate of 175° C s^−1^ and a cooling rate of −125° C s^−1^ with a thermal time constant of 0.27 s.

In this work, we propose a four-tether Silicon MicroThermoCycler (SMTC) structure, which is more robust in terms of mechanical stability than a similar three-tether TC structure and still preserves the comparable thermal insulating properties of the latter. Furthermore, the proposed approach employs titanium heater resistors and two-wire RTDs, enabling the realization of high electrical resistances within a small footprint. This approach has the potential to reduce the device size without compromising RTD sensitivity, RTD leads resistance errors, or heater lead power losses, making it highly suitable for use in Point-Of-Care (POC) PCR analytics.

## 2. Design

**Figure 1:**
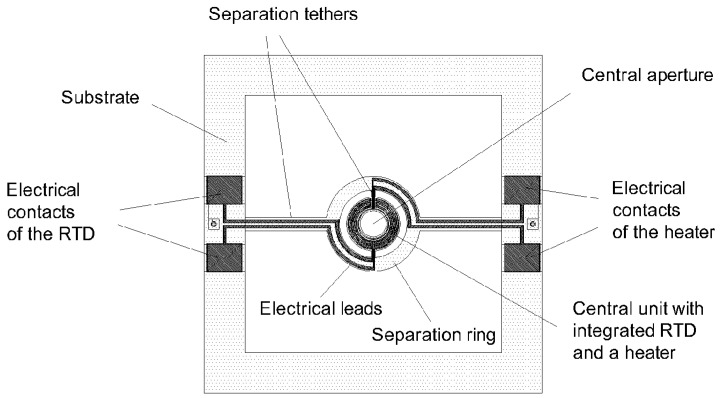
Schematic diagram of a hollow structure SMTC from the bottom side.

Figure 1 shows a schematic diagram of a proposed silicon SMTC. The SMTC comprises a thin film heater and a Resistance Temperature Detector (RTD), both made from titanium and integrated into the bottom of the central unit. The central unit is in the shape of a disc (solid structure) or in the shape of circular annuli (hollow structure). The solid structure allows for the observation of the fluorescence response by exciting the molecules from the top side, while the hollow structure enables the observation of the fluorescence response by exciting the molecules from the bottom side of the SMTC chip.

The central unit is mechanically and electrically attached to the surrounding substrate by four tethers and a separation ring (annular cylinder shape) to provide electrical connections and thermal separation from a substrate. A substrate comprises electrical contacts for a heater and an RTD to enable an SMTC to be connected to a Printed Circuit Board (PCB) by soldering or gluing with conductive solder paste.

In order to reduce the power consumption of a TC (a requirement for portable POC systems), adequate thermal separation of the central unit from the substrate should be ensured. Adequate thermal separation can be provided by the proper design of separating tethers and a separation ring, which should be as narrow as possible. However, narrow tethers and a separation ring allow only a limited number of low-resistance electrical connections to the central unit. Therefore, in the proposed structure, two-electrode RTDs have an apparent advantage over four-electrode RTDs. To minimize measurement error in two-electrode RTD measurements, the resistance of the RTD should be significantly higher than the resistance of the leads. With this approach, the impact of lead resistance, due to factors such as the length and type of measuring leads between the RTD and the instrument, is minimized. A prevalent material for the fabrication of the microheater and the RTD thin film is platinum. Because of its low sheet resistivity, which is at least three times lower than, e.g., titanium’s sheet resistivity [26], it is difficult to obtain high resistance values on a small footprint. Furthermore, platinum is an expensive material, is more difficult to process, requires an additional prime adhesive layer (Cr or Ti), and exhibits a lower Temperature Coefficient of Resistance (*TC_R_*) than, e.g., nickel or titanium [27].

In this study, titanium thin film heater resistors and two-wire RTDs were designed and fabricated. Substituting the commonly employed platinum with titanium in the fabrication of thin film heaters and RTDs enabled the realization of a small device with high heater and RTD electrical resistances on a small footprint, yielding 98 Ω and 725 Ω, respectively, with a high *TC_R_* of 4395 ppm/°C. Scaling down a device without reducing the electrical resistance prevented additional RTD measurement errors due to the lead resistance effect in two-electrode measuring. Furthermore, because of the preserved relatively high heater resistance, power losses in the heater leads were mitigated. The electrical resistance of electrical connections and contact pads was additionally reduced by applying an Ag layer. A heater and an RTD were electrically insulated from a Si substrate and surface protected with thermal oxide and plasma-enhanced silicon nitride (PECVD SiN).

The proposed SMTC structure was specifically designed for use with a disposable reaction chamber. In this setup, the analyte, typically a liquid reagent containing the sample in a volume of 0.5 µL, is pipetted onto the glass disc positioned on top of the SMTC chip. It is then covered with a drop of mineral oil to prevent evaporation of the analyte. The primary benefit of this disposable approach is its ability to prevent cross-contamination between samples.

The geometry and dimensions of the SMTC chip were set as follows: The Si substrate was in the shape of a concentric square annulus with inner and outer square side lengths of 19 mm and 25 mm, respectively. This chip size was chosen to ensure safe and convenient handling, which includes the placement and removal of the disposable reaction chamber (glass disc). This size also allows for the fabrication of five chips on a four-inch silicon wafer. The length of the tethers connecting the central part of the SMTC to the insulation ring and further to the substrate were set to 6 mm and 0.5 mm, respectively. The diameter of the SMTC central unit was set to 4 mm. This dimension was chosen based on the minimal sample volume (0.5 µL) specified by our application.

Crucial design parameters, i.e., the thickness of the silicon substrate (*T_Si_*), the width of the inner tether (*w_t_*_i_), the width of the outer tether (*w_to_*), the width of the separation ring (*w_r_*), and the diameter of the central aperture (*d_a_*), were first investigated by numerical simulations to determine the influence of these parameters on the mechanical and thermal characteristics of the device. This approach facilitated a final determination of the structure geometry prior to SMTC prototype fabrication.

## 3. Analytical Modeling

To gain a detailed insight into the SMTC’s operation, it was essential to obtain the relevant physical background, which allowed us to study the temperature response of the SMTC under electrical excitation. For this purpose, it was reasonable to introduce an analytical model of SMTC heating and cooling. The analytical model included two essential system parameters, i.e., the thermal conductance G and the thermal capacitance H. The exact values of both parameters for a given system can be derived from the results of the measured or FEM-simulated temperature response of the system by fitting the temperature response characteristic curve to the analytical model, as will be further demonstrated below.

### 3.1. Analytical Model of Heating

As our characterization setup was limited to the constant current driving of the SMTC heater, this type of excitation was also introduced in the analytical SMTC model.

A thermal step response of an SMTC to the electrical current I flowing through a heater meander can be described analytically by the heat balance differential equation:

(1) $$G\Delta T+H\frac{d\Delta T}{dt}=I^{2}R_{20}(1+TC_{R}\Delta T)$$

where $G\left[W/K\right]$ denotes the thermal conductance from the SMTC central structure (with the integrated heater and the RTD) to the ambient (heat sink), $H\left[J/K\right]$ is the thermal capacitance of the SMTC central structure (the product of the mass and its specific heat), $R_{20}$ is the heater resistance at the temperature of 20 °C, $TC_{R}$ is the temperature coefficient of a heater resistance, and ΔT is the difference between a heater temperature and the ambient temperature of 20 °C $\left(\Delta T=T-T_{a}\right)$.

Equation (1) can be arranged into a standard form of a first-order linear non-homogeneous differential equation as follows:

(2) $$\Delta T+\frac{H}{G-I^{2}R_{20}TC_{R}}\frac{d\Delta T}{dt}=\frac{I^{2}R_{20}}{G-I^{2}R_{20}TC_{R}}$$

and solved for the boundary condition $T(0)=T_{a}$ as follows:

(3) $$T(t)=T_{a}+\frac{I^{2}R_{20}}{G-I^{2}R_{20}TC_{R}}\left(1-e^{-\frac{G-I^{2}R_{20}TC_{R}}{H}t}\right)$$

As indicated by Equation (3), the heater temperature at a steady state is a function of the heater current I, the heater resistance at ambient temperature $R_{20}$, the temperature coefficient of the heater resistance $TC_{R}$, and the thermal conductance G between the heater and the ambient:

(4) $$T_{\infty }=T_{a}+\frac{I^{2}R_{20}}{G-I^{2}R_{20}TC_{R}}$$

On the other hand, the thermal time constant of the system τ is a function of the heater current I, the heater resistance $R_{20}$, the temperature coefficient of heater resistance $TC_{R}$, and both thermal constants, i.e., thermal conductance G and a thermal capacitance H:

(5) $$\tau =\frac{H}{G-I^{2}R_{20}TC_{R}}$$

If Equation (5) were simplified and the relative change in resistance due to temperature change were neglected $\left(TC_{R}=0\right)$, the time constant τ would be equal to H/G and therefore independent of the current through the heater I. However, in this case, the current through the heater I would still impact the final temperature reached by the heater.

In order to obtain the thermal constants G and H of the SMTC system, the general solution of the heat balance differential Equation (2), expressed as $T(t)=T_{0}+a(1-e^{-bt})$, can be fitted to the simulated or measured temperature response of the system. The thermal constants G and H can then be derived from the coefficients a and b, which are determined by curve fitting as follows:

(6) $$G=I^{2}R_{20}\left(\frac{1}{a}+TC_{R}\right)$$

(7) $$H=\frac{G-I^{2}R_{20}TC_{R}}{b}$$

The analytical heating equation (Equation (3)) shows that the rate of heating from the initial temperature to the steady-state temperature can be controlled by adjusting the current through the heater I (Equation (5)), and, more importantly, this current can influence the final steady-state temperature, potentially exceeding the target temperature. As a result, a higher initial current enables the SMTC to reach the target temperature faster than within 5τ. This approach, involving a higher initial current than necessary to reach the target temperature as quickly as possible and without overshooting, employs a Proportional–Integral–Derivative (PID) feedback controller with carefully optimized *P*, *I*, and *D* coefficients. Although this will be the operating mode of the proposed SMTC, it is beyond the scope of this work.

For effective heating, it is crucial to minimize the thermal conductance G so that, for a given current, the final temperature is as high as possible (Equation (4)). This is important for the energy efficiency required in POC analysis systems. Additionally, minimizing the thermal capacitance H is essential to ensure that the time constant τ is as small as possible (Equation (5)), which will accelerate the cooling of the SMTC system.

### 3.2. Analytical Model of Cooling

The cooling analytical model of the SMTC is similar to the heating analytical model (Equation (1)), except that the cooling the electrical power of the heater is set to zero:

(8) $$G\Delta T+H\frac{d\Delta T}{dt}=0$$

Equation (8) is arranged into a standard form of a first-order linear homogeneous differential equation as follows:

(9) $$\Delta T+\frac{H}{G}\frac{d\Delta T}{dt}=0$$

and solved for the initial boundary condition of ΔT(0)=T, where $\Delta T=T-T_{a}$.

The general solution of Equation (9) is given as follows:

(10) $$T(t)=T_{a}+Ae^{-\frac{G}{H}t}\to A=T-T_{a}\;T(t)=T_{a}+(T-T_{a})e^{-\frac{G}{H}t}$$

From the analytical equation of cooling, it can be deduced that the cooling rate depends solely on the thermal conductance G and the thermal capacitance H of the heater. Since the time constant is τ=H/G, the cooling time to room temperature can be estimated as $t_{cool}=5\cdot H/G$. In practice, in PCR thermo-amplification, the sample is not cooled to room temperature in an individual cycle but typically from 95 °C (denaturation) to the process of annealing, which occurs at around 55 °C [3]. Therefore, the cooling time is faster but still depends on the crucial thermal parameters G and H. Although a well-designed temperature regulator can speed up the heating, the cooling rate depends solely on the thermal parameters of the system. For cooling, it would be desirable to minimize the thermal capacitance of the heater while maximizing the thermal conductance from the heater to the silicon substrate and surroundings. Such a structure, with a small thermal conductance *G* and a large thermal capacitance *H*, cannot be realized in practice since the parameters *G* and *H* are interdependent, as will be demonstrated in Section 7.1.2.

## 4. FEM Numerical Modeling

The previous section described the analytical models for heating and cooling an SMTC system when a constant heater current was applied. Unfortunately, the analytical model has its drawbacks since, in order to compute the temperature response of the system, it is necessary to know both the thermal conductance of the heater *G* and the thermal capacitance of the heater *H*. These two parameters depend on the geometry of the structure itself and on the material and thermal properties of individual components such as their heat capacity, thermal conductivity, material density and heat transfer to ambient via convection and conduction. The latter should be given by an associated heat transfer coefficient. Therefore, numerical simulations by FEM in the Comsol Multiphysics 5.2 simulation environment were additionally applied. Moreover, this simulation software enables the study of the mechanical strength of the system, which was an important guide in the design of the structure.

### 4.1. Mechanical Simulations

**Figure 2:**
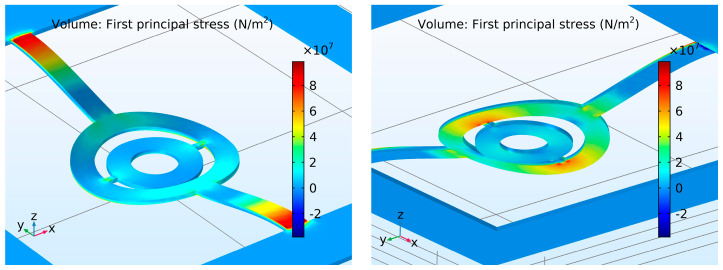
Four regions in the final SMTC structure (see the last paragraph in Section 7.1.2) where the simulated tensile stress due to a downward load is the highest. View from the top (**left**) and view from the bottom (**right**).

In order to investigate the influence of the crucial design parameters on the mechanical strength of the SMTC structure, numerical mechanical strength analysis was initially employed. When the tensile stress in the structure exceeds the tensile strength of the silicon (tensile strength is between 165 and 180 MPa [28]), the structure breaks. Three crucial design parameters were varied one at the time through a specified range by using a parametric sweep procedure defined by Comsol. The model was discretized using a tetrahedral mesh with a maximum element size of 1500 µm to ensure sufficient resolution. Second-order tetrahedral elements were employed in the simulation. A stationary fully coupled MUMPS direct solver was utilized, with a relative tolerance of 1 × 10^−3^, to accurately capture the stationary behavior of the system. Silicon was defined as an isotropic linear elastic material. The bottom surface of the substrate was defined as “fixed constrain boundary”, while the upper surface of the central unit was defined as the “boundary load” function in the mechanical module of Comsol. To compute the maximum tensile stress, “derived values” in Comsol were employed, where the “volume maximum” functionality was applied to compute the maximum tensile stress in the structure. The load was increased until the tensile stress reached the critical failure stress of silicon. The tensile stress was computed in Comsol as the “first principal stress”. The critical points where the tensile stress values were the highest were determined in the simulation model. These points were found to be in the surroundings of the tethers, so the focus was on the contribution of the tether geometry to the mechanical strength of the structure. The tensile stress was the highest on the upper sides of the outer tethers, just at the attachment to the Si substrate, and on the bottom sides of the inner tethers, just at the attachment to the separation ring. This defines the places where the SMTC will break under a downward load (Figure 2).

### 4.2. Thermal Simulations

The 3-D SMTC thermal model was built in the Comsol simulation environment. The thermal behavior of the SMTC can be explained by considering only one physical model. The device is locally heated by a biasing current. The generated heat is dissipated through the device by heat conduction (Comsol module: Heat Transfer in Solids). In addition, SMTC boundaries in contact with ambient air are employed in the boundary convective heat flux in Comsol, which describes the heat dissipation out of the chip.

A brief description of the relevant equations, boundary conditions, material parameters, and a solution strategy is given below.

The heat equation is an energy balance equation deduced from the first law of thermodynamics. For solids, it takes the following form when formulated on the spatial frame:

(11) $$\rho C_{p}\frac{\partial T}{\partial t}+\nabla q=Q$$

where T is the temperature, ρ is the solid density, $C_{p}$ is the solid heat capacity at constant pressure, q is the heat flux vector, and Q is the heat source (or sink).

The thermal conductivity k describes the relationship between the heat flux vector q and the temperature gradient ∇T, which is a Fourier’s law of heat conduction:

(12) q=−k∇T

Heat dissipation out of the chip was defined by a boundary heat flux node (part of the model in Comsol). The heat flux $q_{0}$ was set to convective heat flux as $q_{0}=h(T_{ext}-T)$, where h is a heat transfer coefficient from the SMTC to the surroundings and $T_{ext}$ is a temperature of surrounding air.

The dissipated heat flowrate from the heater positioned on the bottom surface of the central unit was defined as the “Boundary heat source” function in Comsol. The heat flowrate of the boundary source was computed by squaring the current passing through the heater and multiplying it by the electrical resistance of the heater. The electrical resistance was derived from the heater temperature T, the heater resistance at 20 °C $R_{20}$, and the heater temperature coefficient of resistance $TC_{R}$. To compute the SMTC heater temperature, the “Derived values” and “Surface average” functionalities in Comsol were employed.

The transient simulation was solved in the time range from 0 to 200 s with a time step of 0.1 s. The time-dependent fully coupled PARDISO solver with a relative tolerance of 0.01 was selected.

The reference temperature of solid structures was set to 293.15 K. Essential material parameters were taken from a Comsol library and are listed in Table 1 for the temperature of 300 K.

In order to compute the contribution of the crucial design parameters to the thermal properties of the SMTC (thermal conductance of the heater to the surroundings *G* and the heat capacitance of the heater *H*), a transient simulation was performed for selected parameter values to obtain the temperature response of the heater for default heater current of 50 mA. The *G* and *H* values were then obtained by curve fitting in data analysis and graphing software by means of fitting an analytical model of heating (Equation (9)) to the simulated temperature response of the heater. In addition to the determination of the thermal parameters *G* and *H*, the well-fitted curves with the coefficient of determination Rsqr < 0.99 confirmed the appropriateness of the proposed analytical model.

To be able to automatically sweep the selected values of the analyzed parameter over the desired range, the “Parametric sweep” function in Comsol was employed. The additional simulation results revealed that the temperature of the top surface of the SMTC central unit differs from the heater temperature by less than 0.02 °C at the heater current of 60 mA.

**Figure 3:**
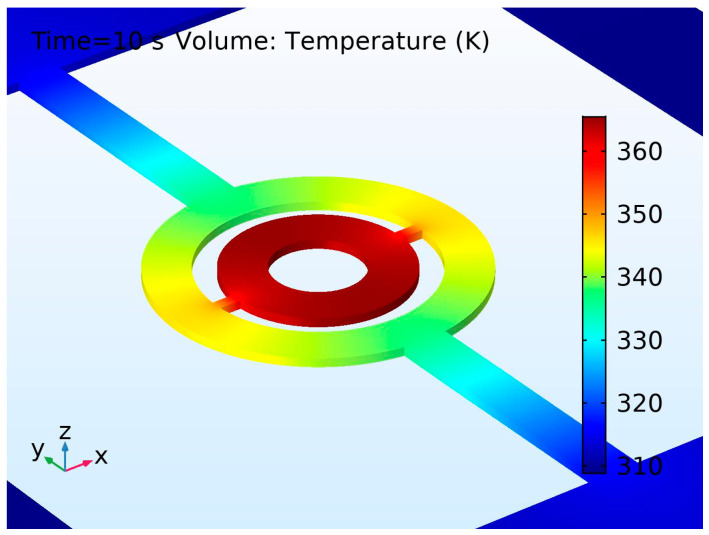
Simulated temperature distribution in the final SMTC structure (see the last paragraph in Section 7.1.2), revealing the efficiency of the tethers and separation ring in terms of thermal separation of a central unit from a substrate.

A simulated temperature distribution in the SMTC, revealing the efficiency of the tethers and separation ring in terms of thermal separation of a central unit from a substrate, is shown in Figure 3.

## 5. Fabrication

**Figure 4:**
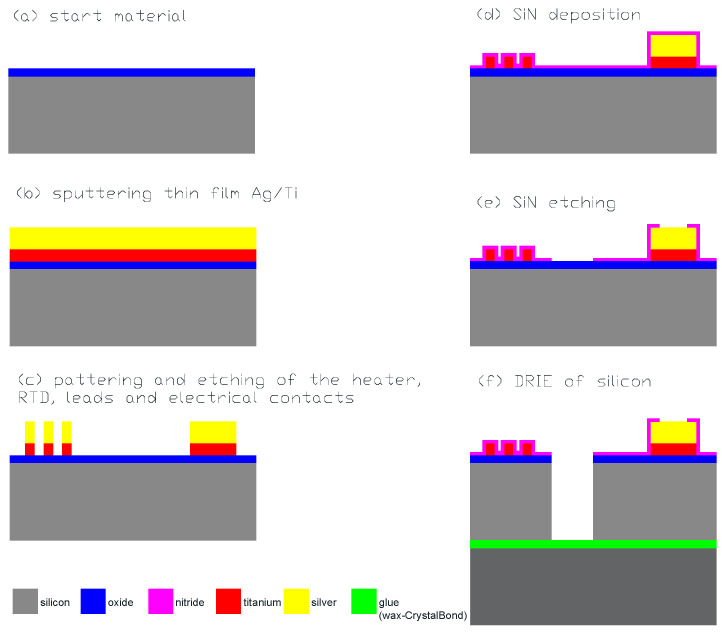
SMTC fabrication process scheme.

The fabrication scheme with the most essential fabrication steps is shown in Figure 4. The full description of the fabrication process is found under the Supplementary Materials. A short description of the fabrication process follows.

The process started with the growth of a 0.35 µm thick thermal oxide on a 100 mm diameter silicon wafer with a thickness of 200 µm and resistivity of 15 Ωcm. This layer provides an electrical isolation of the heater, RTD, and connections from the conductive silicon substrate.

**Figure 5:**
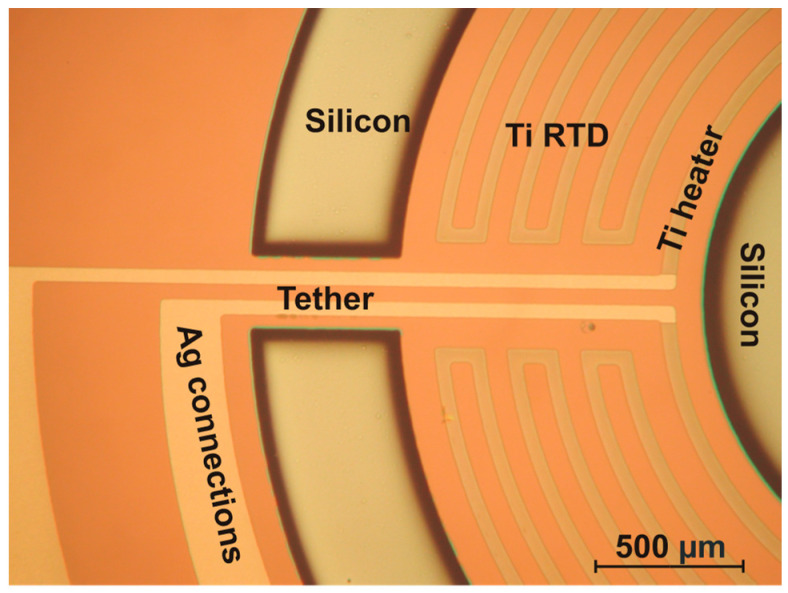
MSTC fabrication detail showing the connection lines of the Ti heater, the resistive meanders of the Ti RTD and Ti heater, and the partially etched silicon region of the tether and central aperture, etched to a depth of 50 µm.

The next step was the physical deposition of a thin film metal sandwich of titanium and silver by DC magnetron sputtering with thicknesses of 780 nm and 800 nm, respectively, to fabricate the heater, RTD sensors, and electrical connections. These devices were formed by photoresist patterning followed by selective wet etching of these layers. Then, the electrical devices were protected by plasma-deposited 300 nm thick Sin, patterned and dry etched to open the contact pads and areas for through silicon DRIE etching. After resist patterning and before DRIE of silicon, a dummy wafer using wax grease was bonded by processing a wafer, enabling through-wafer etching. Namely, good thermal contact was necessary for a successful vertical etch through the defined silicon structure. Before DRIE etching using the Bosch process technique, the plasma etching of the thermal oxide was performed in the same system. Figure 5 shows the connection lines of the Ti heater, the resistive meanders of the Ti RTD and Ti heater, and the partially etched silicon region of the tether and central aperture, etched to a depth of 50 µm. The image was captured during the through-silicon DRIE etching process, at a stage where the etch rate was measured.

## 6. Electrical Characterization

**Figure 6:**
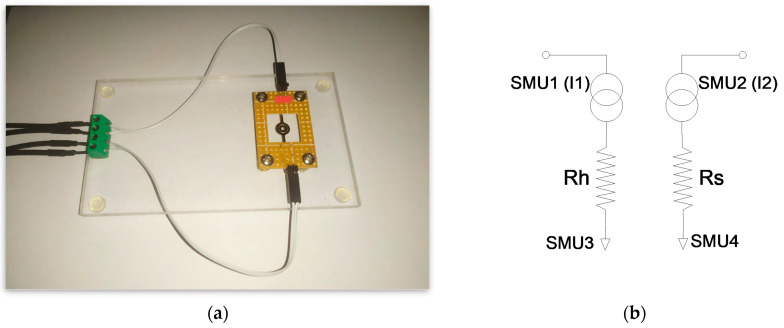
The main board with the supporting PCB board and the SMTC chip (**a**). The measurement configuration of a semiconductor parametric analyzer (**b**).

In order to ensure the mechanical separation of fragile SMTC chips from bulky measuring cables, a corresponding PCB support board (45 mm × 30 mm) was provided for each SMTC chip. Electrical connectivity was ensured via two electrical connectors, internal copper leads, and four thin copper wires (Φ = 130 µm), which, on its other side, contacted the electrical pads of the SMTC chip. The main board was cut from acrylic glass (80 mm × 140 mm × 5 mm). The massive design allowed the SMTC chips to be mounted stably via corresponding PCB support boards and facilitated the handling of the device (Figure 6a).

A semiconductor parametric analyzer, HP4155A (Agilent Technologies, Santa Clara, CA, USA), was employed for electrical characterization. The heater was connected to SMU1, and the RTD to SMU2, with both channels defined as current sources (Figure 6b). A measuring current of 100 µA was set for the RTD, ensuring minimal self-heating of the sensor. The grounding of the heater and RTD by using separate SMUs (SMU3 and SMU4) channels improved the precise measurement of RTD voltage because the serial voltage drop in this chain was minimized. Currents I1 and I2 were applied by SMU1 and SMU2, and voltage was measured on each channel relative to the ground to calculate the power of the heater and RTD resistance.

## 7. Results

Section 7.1 describes the numerical simulations conducted in order to determine the influence of crucial design parameters on the SMTC’s mechanical strength and essential thermal properties, i.e., thermal conductance *G*, thermal capacitance *H*, and time constant τ. Based on the obtained simulation results, crucial design parameter values (crucial design parameters are listed in Section 2) were set, and afterward, the prototype devices were fabricated and characterized. In Section 7.2, the characterization results are given.

### 7.1. Investigation of Crucial SMTC Design Parameters by Numerical Simulations

The mechanical strength of an SMTC is an important property that ensures the safe and robust handling of the device. The proposed SMTC is intended to be used with a disposable reaction chamber in the form of a glass disc, which the operator will attach and detach from the central unit of the SMTC by using tools such as tweezers or a loading arm. A particular challenge lies in the selection of the crucial design parameter values to make the structure adequately mechanically robust while minimizing both the thermal conductance *G* from the central unit of the SMTC to the ambient and the thermal capacitance *H* of the SMTC.

#### 7.1.1. Structural Mechanical Simulations

The influence of three crucial design parameters on the mechanical strength of an SMTC, i.e., the inner tether width *w_ti_*, the outer tether width *w_to_*, and the silicon substrate thickness *t_Si_*, was studied by means of numerical simulations performed in Comsol. A detailed description of the simulation model and the simulation strategies are described in Section 4.1. The simulated ranges of crucial design parameters in structural mechanical simulations are given in Table 2. The default parameter values for the silicon substrate thickness, the separation ring width, and the central aperture diameter were set to 200 µm, 1 mm, and 2 mm, respectively.

**Figure 7:**
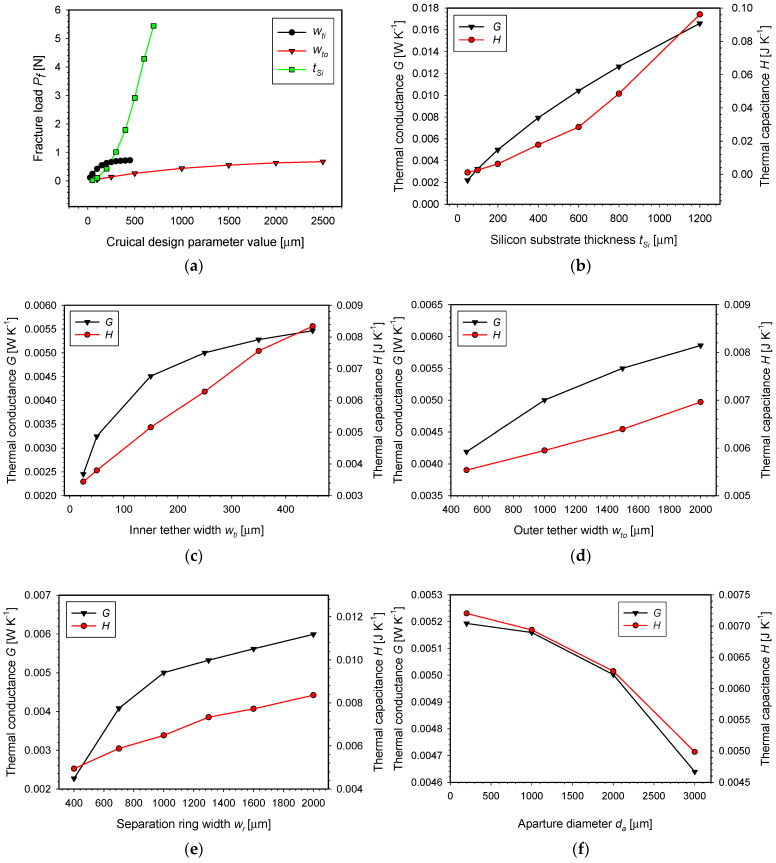
Influence of crucial design parameters on the (**a**) mechanical strength and (**b**–**f**) thermal properties of the SMTC.

First, the inner tether width was varied from 25 µm to 450 µm (Figure 7a), keeping the silicon substrate thickness, the separation ring width, and the central aperture diameter at default values.

In order to compute the SMTC fracture load as a function of the inner tether width, it was necessary to ensure that the maximum tensile stress in the SMTC structure would indeed be in the region of the inner tether and not the outer tether. Therefore, the width of the outer tether was temporarily set to 3 mm, which shifted the maximum tensile stress to the inner tether. In the lower range (between 25 µm and 250 µm of width), the fracture load vs. inner tether width function takes the form of an exponential rise to maximum function, and in the upper range (between 300 and 450 µm of width), it takes the form of a linear function with a slope coefficient of 0.024 N µm^−1^. In order to ensure the adequate mechanical strength of the fabricated structure, a value of 250 µm was chosen for the inner tether width parameter, which provided an SMTC fracture load of 0.66 N (downward load on the central unit). According to the simulation results, a lower value of the parameter would weaken the structure considerably, while a higher value would not significantly increase the mechanical strength of the structure. In addition, this was about the minimum width of the tether, limited by our fabrication process, through which the appropriate electrical connections to the heater and RTD could be routed.

In the next step, the mechanical strength of the structure in relation to the outer tether width parameter value was studied. The outer tether width was varied from 100 µm to 2500 µm (Figure 7a), keeping the silicon substrate thickness, the separation ring width, and the central aperture diameter at default values, as defined in the first paragraph. In this simulation, the inner tether width was set to 2 mm to ensure that the maximum tensile stress in the structure was in the region of the outer tether. The response of the simulated fracture load on the SMTC central unit could be mathematically described by the exponential rise to maximum function, which, in the initial part between 0 and 1000 µm, could also be approximated by a linear function with a slope coefficient of 450 N m^−1^. For the outer tether width parameter, a value of 1 mm was selected to ensure sufficient mechanical strength of the structure (0.45 N). By increasing only the width of the inner and outer tethers, a maximum SMTC mechanical strength of 0.7 N can be assured. In order to further improve the strength of the structure, it would be necessary to also increase the thickness of the silicon substrate.

Therefore, in the following step, the silicon substrate thickness was varied from 50 µm to 700 µm (Figure 7a), keeping the separation ring width and central aperture diameter at default values, as defined in the first paragraph, and the inner and outer tether width at 250 µm and 1000 µm, respectively. The characteristic of a fracture load vs. silicon substrate thickness is an exponentially increasing function. If a maximum fracture load of 0.43 N is achieved at a 200 µm thickness, a doubling of the thickness from 200 µm to 400 µm can provide as much as four times the initial fracture load.

To determine how our improved four-tether design compares to the conventional three-tether TC structure in terms of mechanical strength, we compared the simulation results of our proposed design with the results of the modified design with one of its external tethers removed. The simulation results indicated that the four-tether design exhibited 460% greater mechanical strength than the three-tether configuration. Specifically, the four-tether design demonstrated a fracture load of 0.427 N, while the modified three-tether design yielded a fracture load of 0.092 N.

The simulation results suggest that structural mechanical strength can be ensured by a sufficient thickness of a silicon substrate and by a sufficient width of inner and outer tethers. An increase in these parameter values improves the mechanical strength of the structure; however, at the same time, it deteriorates the thermal properties of the structure. Therefore, further thermal numerical simulations were performed to investigate the effects of the crucial design parameters on the thermal properties of the SMTC.

#### 7.1.2. Thermal Simulations

The influence of five crucial design parameters on the thermal properties of the SMTC, i.e., the silicon substrate thickness *t_Si_*, the inner tether width *w_ti_*, the outer tether width *w_to_*, the separation ring width *w_r_*, and the central aperture diameter *d_a_*, was studied by numerical simulations.

The thermal conductance *G* and the thermal capacitance *H* were computed from the simulated thermal SMTC response for a default heater current of 50 mA. A detailed description of the simulation model, solving strategy, and curve fitting method are presented in Section 4.2. The simulated ranges of crucial design parameters in thermal simulations are given in Table 3.

In the first step, the influence of the silicon substrate thickness on the thermal properties of the SMTC structure was analyzed (Figure 7b). In Section 7.1.1, it was found that the thickness of the silicon substrate is a crucial design parameter that significantly affects the mechanical properties of the SMTC. Similarly, the results of the thermal simulations confirm that this design parameter has a strong impact on both the thermal conductance *G* and the thermal capacitance *H*. Both thermal parameters are positively correlated, meaning that an increase in the thickness of the substrate results in a simultaneous increase in both thermal parameters *G* and *H*. The dependencies of *G* and *H* can be approximated with linear functions. For thicknesses up to 600 µm, the slope coefficients are 1.48 × 10^−5^ W·K^−1^·µm^−1^ and 5.08·× 10^−5^ J·K^−1^·µm^−1^, respectively. For thicknesses between 600 µm and 1200 µm, the slope coefficients are 1.02·× 10^−5^ W·K^−1^·µm^−1^ and 11.38·× 10^−5^ J·K^−1^·µm^−1^, respectively.

The thickness of the silicon substrate was chosen to be 200 µm, as this provided the structure with favorable thermal properties and satisfactory mechanical strength. Additionally, we have extensive experience employing 200 µm silicon wafers in various MEMS structures, making it a reliable choice for using them in our SMTC fabrication process.

In the second step, the inner tether width was varied from 25 µm to 450 µm, keeping the silicon substrate thickness, the separation ring width, the central aperture diameter, and the outer tether width at selected default values of 200 µm, 1 mm, 2 mm, and 1 mm, respectively (Figure 7c).

The characteristic describing the thermal conductance *G* as a function of the inner tether width resembles an exponential rise to maximum function, while the characteristic of thermal capacitance *H* is a linear function with a slope coefficient of 1.153 × 10^−5^ J·µm·K^−1^. For the effective thermal isolation of the heater from the surroundings, i.e., to maintain a low thermal conductance *G*, it is essential to minimize the tether width parameter value. However, based on the *G* characteristics, increasing the width beyond 200 µm does not significantly increase the thermal conductance *G*, though it does impact thermal capacitance *H.*

In the third step, the outer tether width parameter was varied from 500 to 2000 µm (Figure 7d). The responses of both thermal parameters are similar in shape to those observed by the variation in the inner tether width but are less steep. Compared with the inner tether width parameter characteristics, the thermal conductivity function *G* resembles an exponential rise to a maximum, while the thermal capacitance *H* is a linear function with a flatter slope coefficient of 9.375 × 10^−7^ J·µm·K^−1^. The results indicate that decreasing the inner tether width to less than 250 µm has a much greater impact on the thermal properties of the SMTC than varying the outer tether width across the simulation set.

A width of 1000 µm was selected for this design parameter value to ensure sufficient mechanical strength of the structure (Section 7.1.1).

In the fourth step, a separation ring parameter was varied from 400 µm to 2000 µm (Figure 7e) while maintaining the silicon substrate thickness, central aperture diameter, and tether width at their default values. The thermal conductance *G* and thermal capacitance *H* characteristics are very similar to those observed at inner tether or outer tether width parameter variations. Specifically, the thermal conductance *G* initially exhibits an exponential rise to a maximum within the interval of 400 µm to 1300 µm and then transitions to a linear function with a slope coefficient of 9.56 × 10^−7^ W·K^−1^·µm^−1^. The thermal capacitance *H* follows an almost linear function with a slope coefficient of 2.121 × 10^−6^ W·µm·K^−1^. A final separation ring width parameter was set to 1000 µm to ensure sufficient mechanical strength of the SMTC.

In the final step, the central aperture diameter was varied from 200 µm to 3000 µm (Figure 7f). Both characteristics *G* and *H* decrease as the aperture diameter increases (*G* = −1.9634 × 10^−7^ W·K^−1^·µm^−1^ and *H* = −7.8617 × 10^−7^ J·K^−1^·µm^−1^). As the aperture enlarges, the surface area for heat dissipation reduces, and the mass of the central part decreases, resulting in a lower thermal conductance *G* and lower thermal capacitance *H*. The final aperture diameter was set to the default value of 2 mm, which was initially selected based on the sample volume specified by our application. This aperture will enable observation of the fluorescence response by exciting the molecules from the bottom side of the SMTC chip.

The results of the SMTC thermal simulations showed that parameters *G* and *H* are interdependent (see Figure 7b–f). This implies that by varying any crucial design parameter of the SMTC structure, both thermal parameters are increased or decreased simultaneously. Therefore, it is not possible to reduce the thermal capacitance without also reducing the thermal conductance.

When designing an SMTC, the design goal is to achieve the lowest possible thermal conductance *G* and the smallest possible thermal capacitance *H* to minimize energy consumption during the heating phase. This is a fundamental requirement for portable POC systems. When a closed-loop temperature controller is used, the heating rate can be significantly faster than what is dictated by the system time constant τ. However, this does not apply to cooling, as the cooling rate is directly constrained by the system time constant τ, which must be minimized. To investigate how crucial design parameters affect the thermal time constant τ, additional research was conducted.

**Figure 8:**
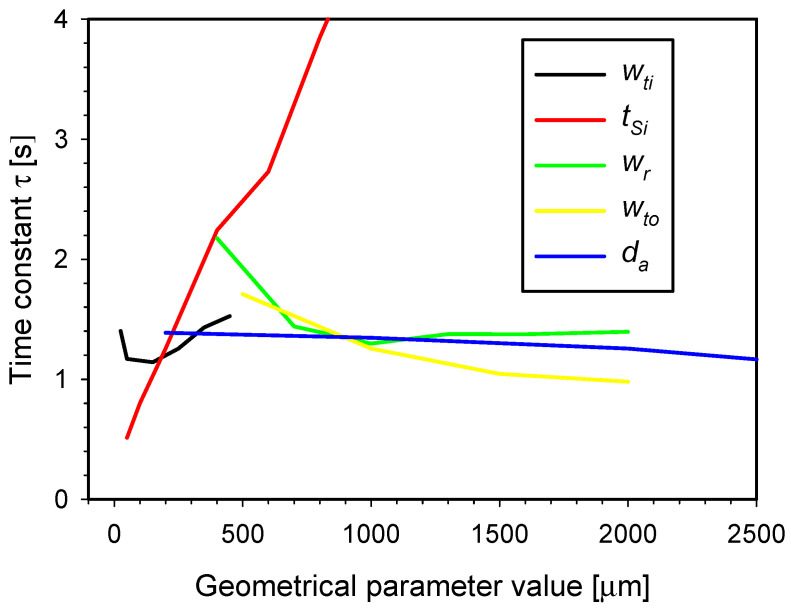
Time constant τ of the SMTC as a function of crucial design parameter values.

Figure 8 shows the characteristics of the time constant τ (τ=H/G) as a function of the crucial design parameter values, calculated based on previous simulations (Figure 7b–f). Attention was given to the three essential parameters that most significantly influence the thermal conductance *G* and thermal capacitance *H*, namely, the silicon substrate thickness, the inner tether width, and the separation ring width. The previous simulation results revealed that increasing the silicon substrate thickness enhances the thermal conductance *G*, with the characteristics resembling an exponential rise to maximum. Because of the improved heat dissipation from the increased Si substrate thickness, a smaller thermal time constant τ and, consequently, faster cooling of the SMTC heater would be expected. However, as the substrate thickness increases, the thermal capacitance *H* of the heater also rises, exhibiting an exponentially increasing trend. This implies that with increasing substrate thickness, the thermal capacitance *H* increases more rapidly than the thermal conductance *G*, ultimately resulting in an increase in the thermal time constant τ as a function of the silicon substrate thickness. The characteristic of the time constant τ is approximately a linear function with a slope of 0.005 s µm^−1^. Therefore, to design a fast SMTC with the smallest possible time constant τ, the Si substrate should be as thin as possible. However, it is crucial to note that reducing the substrate thickness significantly impacts the mechanical strength of the structure (Figure 7a).

The characteristic curve of τ vs. the inner tether width has its minimum of 1.14 s at 150 μm. Variation in the width parameter value upwards or downwards within the simulated parameter range can increase the time constant by up to 33%. During the cooling phase of the SMTC, increasing the tether width enhances the heat dissipation from the central part of the SMTC to the substrate. However, increasing the width also increases the thermal capacitance of the heater, which ultimately results in a slower cooling of the system (higher time constant τ). Based on the simulated characteristic results, it appears that the optimal tether width parameter value would be 150 μm. However, this width would be insufficient to achieve reliable electrical connections. Therefore, a final inner tether width parameter was set to 250 μm.

The characteristic curve of τ for the separation ring width is composed of two linear segments. The first segment shows a decreasing trend up to 700 µm, after which the rate of decrease significantly flattens, and the function remains nearly constant up to the maximum simulated value of 2500 µm. This validates the choice of a parameter value of 1000 µm, as it already approaches the minimum τ. Increasing the width of the separation ring improves the thermal conductance between the heater and the ambient, allowing more heat to transfer through the ring. At the same time, a wider separation ring increases the mass of the system, which slightly raises the thermal capacitance. However, this increase in thermal capacitance *H* does not keep pace with the increase in thermal conductance *G*.

The characteristic curve of τ with respect to the outer tether width shows a slow decline across the simulated range. A parameter value of 1000 µm is appropriate, ensuring that the thermal conductance *G* and the thermal capacitance *H* do not increase excessively. A further reduction in this parameter would lead to an increase in the time constant τ.

The parameter with the least impact on the time constant τ is the aperture diameter. Variations in this parameter result in almost identical changes in both the thermal conductance *G* and the thermal capacitance *H*, maintaining a constant ratio between them.

To determine how our improved four-tether design compares to the conventional three-tether design in terms of thermal properties, we removed one of the outer tethers in the simulation environment and repeated the simulation using our default parameter values. The simulation results showed that the proposed four-tether design yielded 22% greater thermal conductivity than the comparable three-tether design while reducing the system time constant by 57%.

The final values of the crucial design parameters, selected based on the results of mechanical and thermal numerical analysis, are as follows: the silicon substrate thickness was set to 200 µm, the inner tether width to 250 µm, the outer tether width to 1000 µm, the separation ring width to 1000 µm, and the central aperture diameter to 2000 µm.

### 7.2. Characterization Results of the Fabricated Prototypes

**Figure 9:**
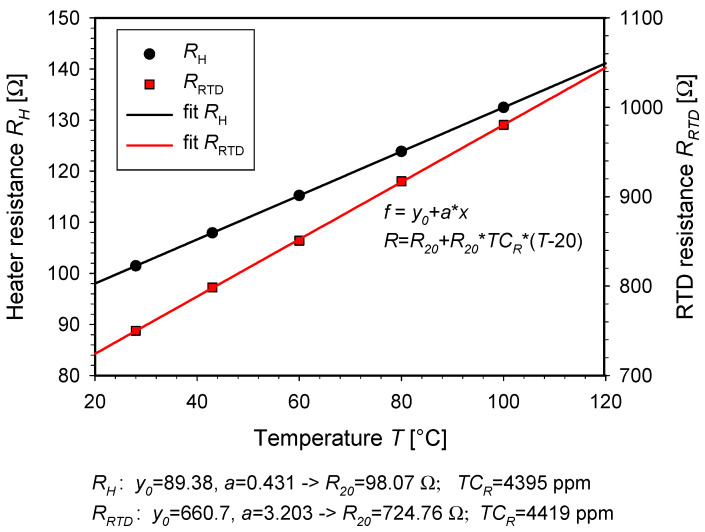
Measured thin film heater and RTD resistance at selected temperatures by heating the SMTC on a laboratory temperature-stabilized heater.

Based on the simulation results (Section 7.1), crucial designed parameter values were set, and a prototype device was fabricated and characterized. The temperature coefficient of the resistance of the fabricated thin film heaters and RTDs was calculated from the measured resistances at selected temperatures by heating the SMTC on a laboratory temperature-stabilized heater (Kika labortechnik typ. RET CV), as shown in Figure 9.

The discrepancy in the measured *TC_R_* coefficient between the heater (4395 ppm/°C) and the RTD (4419 ppm/°C) is 0.5%. This can be attributed to the non-uniformity of the Ti layer across the wafer and measurement error. The measured *TC_R_* value falls within the range of the reported values for Ti thin films found in the literature. Singh B. and Surplice N. A. formed a Ti layer by evaporation onto soda glass microscope slides at room temperature and measured *TC_R_* as a function of thickness [29]. From their review table, which includes their measured values as well as a comprehensive review of *TC_R_* values reported in the literature, it is evident that the *TC_R_* of a thin film titanium layer is highly dependent on both the thickness and the fabrication process (*TC_R_* ranging from −820 ppm/°C to +11,200 ppm/°C). Sing S. et al. deposited a titanium layer by using a DC magnetron sputtering and measured a *TC_R_* of 4146 ppm/°C. In contrast, for an equally deposited platinum (Pt) layer, they measured a *TC_R_* of only 2650 ppm/°C [26].

Two types of SMTC structures were fabricated, differing only in the realization of the central part of the SMTC. The first SMTC type comprises a solid central unit and is defined as a solid structure SMTC (SS). The solid structures allow for the observation of the fluorescence response by exciting the molecules from the top side. The second SMTC type comprises an aperture in the central unit and is defined as a hollow structure SMTC (HS). Hollow structures allow for the observation of the fluorescence response by exciting the molecules from the bottom side of the SMTC chip.

The parametric analyzer HP4155A enabled thermo-electrical characterization of the structure by delivering a constant current through the heater. A detailed description of the characterization process is provided in Section 6. The default current through the heater was set to 50 mA.

**Figure 10:**
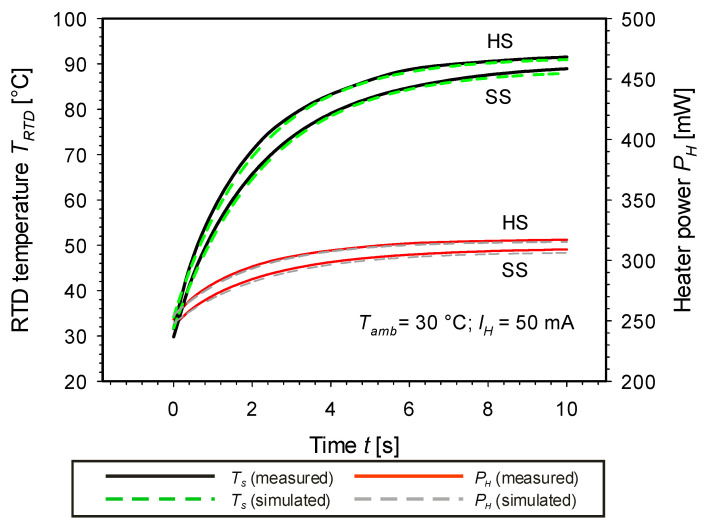
Measured and simulated RTD temperature response and heater power response for HS and SS structures at the default heater current.

The measured RTD temperature and the heater power for HS and SS structures at the default heater current are shown in Figure 10. The characteristics also include the simulated values used to verify the assembled numerical model. The discrepancies between the measurements and simulation can be ascribed to several factors, starting with a non-ideal fabrication process, physical and geometrical model simplifications, selected simulation material parameters, and selected mesh density or solver tolerance.

The temperature response follows an exponential growth function, as it represents the solution to the nonhomogeneous first-order differential equation for heating the SMTC with a constant current (Equation (3)). The results indicate that a current of 50 mA through the heater was sufficient to heat the central part of the SS SMTC from 30 °C to 88 °C in 10 s. At the same time, the electrical power during heating increased in proportion, starting at an initial power of 245 mW and reaching a maximum of 303 mW.

The hollow HS structure, with 11% less thermal capacitance, heats up more quickly and reaches a 4% higher temperature (91.5 °C) while delivering 4.6% higher final electrical power to the heater (317 mW). By means of curve fitting, it is possible to match the solution of the analytical heating model to the measured temperature response. In this way, the thermal conductance *G* and the thermal capacitance *H* were determined for both fabricated structures (Table 4). It was found that the central aperture in the HS structure affects both thermal parameters. In comparison with the solid SS structure, the thermal conductance *G* decreases by 1.8%, while the thermal capacitance *H* decreases by as much as 11%. These findings are consistent with the results of the simulations, which indicate that the thermal conductance *G* decreases by 3.8% because of the 2 mm wide central aperture, while the thermal capacitance decreases by 14% (Figure 7f).

**Figure 11:**
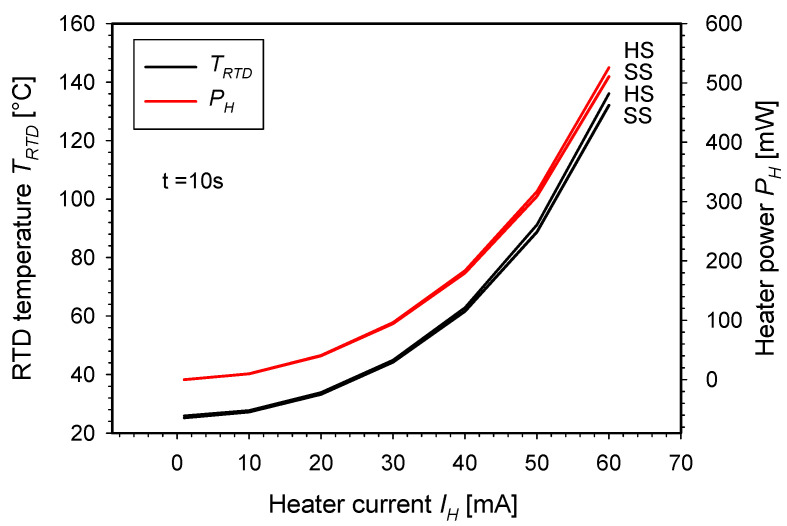
Measured RTD temperature and heater power for HS and SS structures after 10 s for selected applied heater currents.

Following the initial characterization, the temperature response of both structures was further investigated with respect to the set heater current over a period of 10 s (Figure 11). In this measurement, heater currents ranging from 1 mA to 60 mA with a step of 10 mA were applied. As the current through the heater increases, both the RTD temperature reached in 10 s and the power on the heater rise exponentially. The relationship between the temperature and the heater power can be described by an exponential function. The shape of both characteristics in relation to the heater current is identical, as there is a linear relationship between the RTD temperature and the heater power under the assumption of constant current through the heater and a good thermal coupling between the heater and the RTD. It was shown that the current of 60 mA is sufficient to heat the unloaded SS SMTC and unloaded HS SMTC to 132 °C and 134 °C in 10 s at the heater power of 510 mW and 525 mW, respectively.

**Figure 12:**
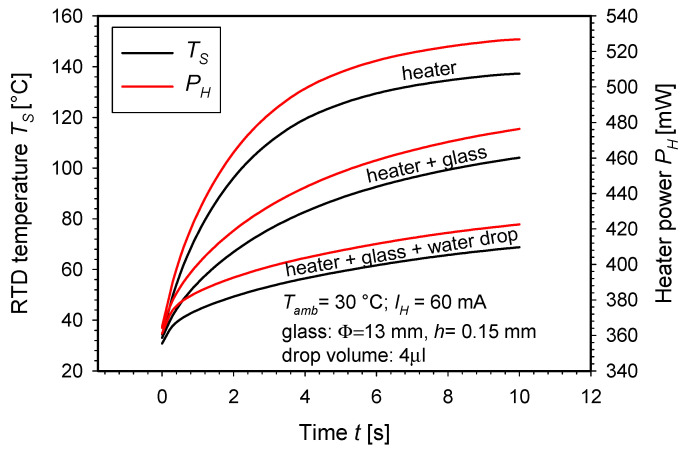
RTD temperature and the heater power vs. time for three different experimental configurations.

Finally, the structures were characterized under actual load conditions. Figure 12 shows the RTD temperature *T_RTD_* and the heater power *P_H_* as a function of time for three different experimental configurations: the heater alone, the heater with an added glass disc, and the heater with an added glass disc and a water drop. The glass has a diameter of 13 mm and a thickness of 0.15 mm, while the volume of the water drop is 4 µL. Measurements were conducted on the HS SMTC structure at an ambient temperature of 30 °C with a constant heater current of 60 mA.

The characteristics of the heater alone show a rapid increase in RTD temperature, reaching 137 °C in 10 s, with the heater power rising to 525 mW. When glass is added, the RTD temperature reaches a lower final value of approximately 103 °C and the final heater power decreases to 475 mW. With the addition of both the glass and the water drop, the RTD temperature further decreases, reaching 68 °C in 10 s, and the heater power is the lowest among the three configurations, ranging to 420 mW.

When glass and water drop were applied, the additional thermal capacitance of the glass and droplet ∆*G* was added to the heater’s thermal capacitance *G*. Similarly, the original thermal conductance *H* was increased by an additional thermal conductance ∆*H*, due to the increased surface area that dissipates the heat into the ambient (i.e., the surface area of the glass and the droplet). Our study focused on optimizing the thermocycler without a load. Adding a load in the simulation environment would shift the simulated characteristics of *G* and *H* (Figure 7b–f) by the constants ∆*G* and ∆*H*, but it would not change their shape, thereby having no crucial impact on the structural optimization process.

## 8. Conclusions

A miniature, fast, and energy-efficient four-tether SMTC for POC PCR analytical systems was proposed. The SMTC comprises a thin-film heater and an RTD, both integrated into the bottom of the central unit. The central unit is mechanically and electrically attached to the surrounding substrate by four tethers and a separation ring to provide electrical connections and thermal separation from a substrate.

In the SMTC fabrication process, platinum, commonly employed in conventional designs, was substituted with titanium. This enabled the realization of smaller heaters and RTDs without reducing their electrical resistance. Scaling down a device without reducing the electrical resistance prevented additional RTD measurement errors due to the lead resistance effect in two-electrode measuring. Furthermore, because of the preserved heater resistance, additional power losses in the heater leads are eliminated.

The device was extensively analyzed through analytical modeling and FEM numerical simulations in COMSOL by using a 3-D thermo-mechanical simulation model. When designing an SMTC suitable for POC PCR applications, the goal is to achieve the lowest possible thermal conductance *G* and the smallest possible thermal capacitance *H* to minimize energy consumption during the heating phase, while simultaneously achieving a minimal time constant τ to increase the cooling rate during the cooling phase of the SMTC. Since these thermal parameters are interrelated, this is not always possible to achieve, and certain compromises must be made during the design process. It has been shown that variation in crucial design parameter values caused the thermal conductance *G* and the capacitance *H* to increase or decrease simultaneously, and the ratio between them determined the time constant τ. Our analysis highlighted the importance of carefully balancing crucial design parameters, such as the silicon thickness and the inner tether width, to optimize the thermocycler performance. It has been shown that minimizing the silicon substrate thickness is critical for reducing energy consumption and reducing the time of thermal cycling (by decreasing essential thermal parameters *G*, *H*, and τ), it must be performed without compromising the mechanical integrity of the device. Likewise, numerical simulations revealed that the four-tether design achieves a 460% improvement in mechanical strength and a 57% reduction in the thermal time constant τ compared with a similar three-tether design, with a trade-off of a 22% increase in heat losses.

Detailed structural and thermal analyses of crucial design parameters guided the optimization of the final geometry, leading to the successful fabrication of prototypes. It was shown that the current of 60 mA was sufficient to heat the fabricated SMTC solid structure and hollow structure to 132 °C and 134 °C in 10 s at the heater power of 510 mW and 525 mW, respectively.

The modified four-tether structure offers a well-rounded solution for point-of-care diagnostic systems, providing a strong combination of mechanical strength, thermal efficiency, and precision in temperature control.

## Figures and Tables

**Table 1:** List of the Si material properties at *T* = 300 K [28] that were applied in the Comsol simulation model.

Density [kg∙m^−3^]	2329
Heat capacity at constant pressure [J∙kg^−1^∙K^−1^]	700
Thermal conductivity [W∙m^−1^∙K^−1^]	120
Tensile strength [MPa]	165
Heat transfer coefficient to surrounding air [W∙m^−2^∙K^−1^]	36

**Table 2:** Simulated ranges of crucial design parameters.

Parameter	Simulated Range
inner tether width *w_ti_*	25 µm–250 µm
outer tether width *w_to_*	100 µm–2500 µm
silicon substrate thickness *t_Si_*	50 µm–700 µm

**Table 3:** Simulated ranges of crucial design parameters.

Parameter	Simulated Range
silicon substrate thickness *t_Si_*	50 µm–1200 µm
inner tether width *w_ti_*	25 µm–450 µm
outer tether width *w_to_*	500 µm–2000 µm
separation ring width *w_r_*	400 µm–2000 µm
aperture diameter *d_a_*	200 µm–3000 µm

**Table 4:** Measured thermal properties for SS and SH structure.

	SS	SH
Thermal conductance *G* [WK^−1^]	0.0056	0.0055
Thermal capacitance *H* [JK^−1^]	0.009	0.008
Thermal time constant τ [s]	1.6	1.46

## Data Availability

The original contributions presented in this study are included in the article/Supplementary Material. Further inquiries can be directed to the corresponding author.

## Supplementary Materials

The following supporting information can be downloaded at: https://www.mdpi.com/article/10.3390/mi15111325/s1, Figure S1: TC fabrication process scheme.

## References

[1] Chadha U., Bhardwaj P., Agarwal R., Rawat P., Agarwal R., Gupta I., Panjwani M., Singh S., Ahuja C., Chakravorty A. (2022). Recent progress and growth in biosensors technology: A critical review. J. Ind. Eng. Chem..

[2] Yeom D., Kim J., Kim S., Ahn S., Choi J., Kim Y., Koo C. (2022). A Thermocycler Using a Chip Resistor Heater and a Glass Microchip for a Portable and Rapid Microchip-Based PCR Device. Micromachines.

[3] Huang B.J., Duang C.L. (2000). System dynamic model and temperature control of a thermoelectric cooler. Int. J. Refrig..

[4] Roy S., Arshad F., Eissa S., Safavieh M., Alattas S.G., Ahmed M.U., Zourob M. (2022). Recent developments towards portable point-of-care diagnostic devices for pathogen detection. Sens. Diagn..

[5] Hajare R., Reddy V., Srikanth R. (2022). MEMS based sensors—A comprehensive review of commonly used fabrication techniques. Mater. Today Proc..

[6] El-Ali J., Perch-Nielsen I.R., Poulsen C.R., Bang D.D., Telleman P., Wolff A. (2004). Simulation and experimental validation of a SU-8 based PCR thermocycler chip with integrated heaters and temperature sensor. Sens. Actuators A Phys..

[7] Ahrberg C.D., Manz A., Chung B.G. (2016). Polymerase chain reaction in microfluidic devices. Lab Chip.

[8] Pal D., Venkataraman V. (2002). A portable battery-operated chip thermocycler based on induction heating. Sens. Actuators A Phys..

[9] Nguyen K.H. (2023). A Rapid and Power-Efficient Laser-Induced Graphene Micro-Thermocycler for DNA Polymerase Chain Reaction. Master’s Thesis.

[10] Just V.M., Welzel F., Jacobs H., Gau G., Hauschultz M.T., Friedo M.H., Foitzik A.H. (2023). Development of a Thermal Cycler for a Low-Cost Real-Time PCR Application. Key Eng. Mater..

[11] Liu W., Warden A., Sun J., Shen G., Ding X. (2018). Simultaneous detection of multiple HPV DNA via bottom-well microfluidic chip within an infra-red PCR platform. Biomicrofluidics.

[12] Wu H., Zhang S., Chen Y., Qian C., Liu Y., Shen H., Wang Z., Ping J., Wu J., Chen H. (2020). Progress in molecular detection with high-speed nucleic acids thermocyclers. J. Pharm. Biomed. Anal..

[13] Lin Y.C., Yang C.C., Huang M.Y. (2000). Simulation and experimental validation of micro polymerase chain reaction chips. Sens. Actuators B Chem..

[14] Dinca M.P., Gheorghe M., Aherne M., Galvin P. (2009). Fast and accurate temperature control of a PCR microsystem with a disposable reactor. J. Micromech. Microeng..

[15] An Y.Q., Huang S.L., Xi B.C., Gong X.L., Ji J.H., Hu Y., Ding Y.-J., Zhang D.-X., Ge S.-X., Xia N.S. (2023). Ultrafast microfluidic PCR thermocycler for nucleic acid amplification. Micromachines.

[16] Mashouf H., Talebjedi B., Tasnim N., Tan M., Alousi S., Pakpour S., Hoorfar M. (2023). Development of a disposable and easy-to-fabricate microfluidic PCR device for DNA amplification. Chem. Eng. Process.-Process Intensif..

[17] Schneegaß I., Köhler J.M. (2001). Flow-through polymerase chain reactions in chip thermocyclers. Rev. Mol. Biotechnol..

[18] Zhang J., Yang Z., Liu L., Zhang T., Hu L., Hu C., Chen H., Ding R., Liu B., Chen C. (2023). Ultrafast nucleic acid detection equipment with silicon-based microfluidic chip. Biosensors.

[19] Ventimiglia G., Pesaturo M., Malcolm A., Petralia S. (2022). A miniaturized silicon lab-on-chip for integrated PCR and hybridization microarray for high multiplexing nucleic acids analysis. Biosensors.

[20] Moore D.F., Syms R.R.A. (1999). Recent developments in micromachined silicon. Electron. Commun. Eng. J..

[21] Nunez-Bajo E., Silva Pinto Collins A., Kasimatis M., Cotur Y., Asfour T., Tanriverdi U., Grell M., Kaisti M., Senesi G., Güder F. (2020). Disposable silicon-based all-in-one micro-qPCR for rapid on-site detection of pathogens. Nat. Commun..

[22] Schneegaß I., Bräutigam R., Köhler J.M. (2001). Miniaturized flow-through PCR with different template types in a silicon chip thermocycler. Lab Chip.

[23] Jie J., Hu S., Liu W., Wei Q., Huang Y., Yuan X., Ren L., Tan M., Yu Y. (2020). Portable and battery-powered PCR device for DNA amplification and fluorescence detection. Sensors.

[24] Daniel J.H., Iqbal S., Millington R.B., Moore D.F., Lowe C.R., Leslie D.L., Lee M., Pearce M.J. (1998). Silicon microchambers for DNA amplification. Sens. Actuators A Phys..

[25] Neuzil P., Zhang C., Pipper J., Oh S., Zhuo L. (2006). Ultra fast miniaturized real-time PCR: 40 cycles in less than six minutes. Nucleic Acids Res..

[26] Singh S., Jejusaria A., Singh J., Vashishath M., Kumar D. (2022). Comparative study of titanium, platinum, and titanium nitride thin films for micro-elecrto mechanical systems (MEMS) based micro-heaters. AIP Adv..

[27] Možek M., Pečar B., Vrtačnik D. (2024). Cost-Efficient Oceanographic Instrument with Microfabricated Sensors for Measuring Conductivity, Temperature and Depth of Seawater. Sensors.

[28] Tilli M., Paulasto-Kröckel M., Petzold M., Theuss H., Motooka T., Lindroos V. (2020). Handbook of Silicon Based MEMS Materials and Technologies.

[29] Singh B., Surplice N.A. (1972). The electrical resistivity and resistance-temperature characteristics of thin titanium films. Thin Solid Film.

