# Supplementary material for: Silicon Microthermocycler for Point-of-Care Analytical Systems: Modeling, Design, and Fabrication
Source: Micromachines (Basel). 2024 Oct 30;15(11):1325. doi: 10.3390/mi15111325 (PMC11596950; doi:10.3390/mi15111325)
Supplement: Supplementary file 1 [file micromachines-15-01325-s001.zip › micromachines-3252604-supplementary.pdf]

Full description of fabrication process:

Silicon TCs were designed and fabricated on double side polished 100 mm silicon wafers, N-type, resistivity of 10 Ω·cm and thickness of 200 µm. The main fabrication steps are presented in Figure 1.

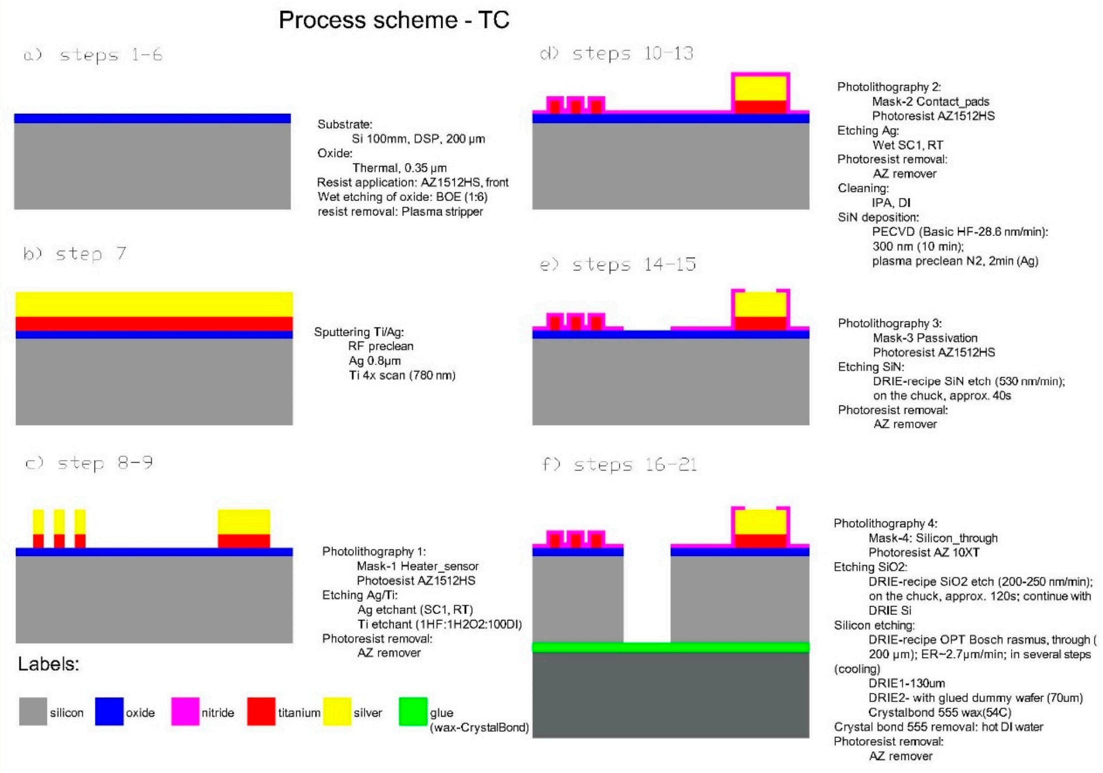

Figure S1. TC fabrication process scheme.

The silicon wafer was first thermally oxidized (1000 °C) to provide an oxide (0.35 µm thick) for the deep reactive ion etching (DRIE) mask in step 18. To remove the oxide on the backside by wet etching in buffered HF (1NH4F:6HF), the front side was protected with AZ 1512HS resist, which was latter removed in an oxygen plasma. The metallization schemes were realized by DC magnetron sputtering using an MRC603 sputtering system. Prior to sputtering, Si wafers were preheated in situ at 180 °C for 3 min, followed by RF pre-cleaning. The working gas pressure of Ar was 0.8 Pa for Ti (purity 99.995%) and 1 Pa for Ag (purity 99.995%). The thickness of the prime Ti and Ag layers was 780 ± 5 nm and 80 ± 2 nm, respectively, measured with a Taylor Hobson surface profilometer.

The heater, RTD, electrical leads, and contact pads were first patterned with AZ1512HS positive photoresist, followed by SC1 wet cleaning at room temperature and an aqueous solution containing hydrofluoric acid (HF) and a hydrogen peroxide solution (H2O2) to etch the Ag and Ti, respectively. The resist was then stripped with AZ 100 remover.

The contact pads were patterned with AZ1512HS photoresist in step 10, followed by SC1 at room temperature to remove the unpattered Ag. After cleaning with IPA and DI, plasma-enhanced chemical vapor deposition (PECVD, HF 28.6 nm/min) of 0.3 µm silicon nitride (SiN) was performed to provide a SiN passivation (protection) layer patterned in step 14 (AZ1512HS,

6 - 8 µm) and etched by deep reactive ion etching (DRIE) in step 15 (35-40s, etch rate of 530 nm min^{-1}).

Etching through a wafer that opens directly onto the electrode during DRIE etching should be avoided. This would cause the temperature control to fail as helium would escape through the holes and could damage the electrode. Therefore, the process was performed by using a dummy wafer bonded to the process wafer with commercial hot water soluble Crystalbond 555 Mounting Adhesive (Aremco Products, Inc., USA). In order to protect the Ag pads from DRIE etching in the following steps, a 10 um thick photoresist AZ 10 XT was patterned in step 16. SiO2 was then etched by DRIE to provide an etch mask for the final step. To allow uniform etching of complex silicon structures over the entire depth of the substrate, a specific OPT Bosch Rasmus process recipe for DRIE etching was developed and applied. In short, etch parameters were set to 15 W and 600 W for platen bias power and coil power, respectively. Helium back side cooling was set to 10 deg °C.

In order is to ensure a mechanical separation of fragile TC chips from bulky measuring cables, a corresponding PCB support board (45 mm x 30 mm) was provided for each TC chip. Electrical connectivity was ensured via two electrical connectors, internal copper leads and four thin copper wires (Φ=130 µm), which on its other side contacted the electrical pads of the TC chip. The PCB board was oriented with the internal electrical connections facing downwards. The TC chip was positioned on the bottom side of the PCB board, likewise orientated with the heater and the RTD structures facing downwards. The chip was attached to the board by using two component epoxy adhesive (UHU plus endfest 300, UHU GmbH & Co., Germany), applied only at the edges of the TC chip. By heating (T > 200°C) and thus disintegrating drops of adhesive (with a soldering tip), the chips could have been post-detached from the associated PCB support board if needed. Each PCB support board comprises four boreholes for attaching it to the main board.

The main board was cut from acrylic glass (80 mm x 140 mm x 5 mm). A massive design allowed the TC chips to be mounted stably via corresponding PCB support boards and facilitated handling of the device. To prevent any unwanted movements of the main board during the measurements, four adhesive anti-slip silicone pads were attached to the bottom side of the board. Electrical connectors on the main board connected the main coaxial measurement cables with the auxiliary measurement cables leading to the attached PCB support board. Furthermore, the main board comprises four metal spacers with internal threads to which the PCB support boards are attached via four screws.
